# miRNAs in Follicular and Oviductal Fluids Support Global DNA Demethylation in Early-Stage Embryos

**DOI:** 10.3390/ijms25115872

**Published:** 2024-05-28

**Authors:** Sogo Aoki, Yuki Inoue, Mao Hamazaki, Shunsuke Hara, Tatsuo Noguchi, Koumei Shirasuna, Hisataka Iwata

**Affiliations:** Department of Animal Science, Graduate School of Agriculture, Tokyo University of Agriculture, Funako 1737, Atsugi 243-0034, Kanagawa, Japan; saw5aoki@gmail.com (S.A.);

**Keywords:** bovine, early embryonic development, extracellular vesicle, follicular fluid, global DNA methylation, miRNA

## Abstract

Global methylation levels differ in in vitro- and in vivo-developed embryos. Follicular fluid (FF) contains extracellular vesicles (EVs) containing miRNAs that affect embryonic development. Here, we examined our hypothesis that components in FF affect global DNA methylation and embryonic development. Oocytes and FF were collected from bovine ovaries. Treatment of zygotes with a low concentration of FF induced global DNA demethylation, improved embryonic development, and reduced DNMT1/3A levels. We show that embryos take up EVs containing labeled miRNA secreted from granulosa cells and the treatment of zygotes with EVs derived from FF reduces global DNA methylation in embryos. Furthermore, the methylation levels of in vitro-developed blastocysts were higher than those of in their vivo counterparts. Based on small RNA-sequencing and in silico analysis, we predicted miR-29b, -199a-3p, and -148a to target DNMTs and to induce DNA demethylation, thereby improving embryonic development. Moreover, among FF from 30 cows, FF with a high content of these miRNAs demethylated more DNA in the embryos than FF with a lower miRNA content. Thus, miRNAs in FF play a role in early embryonic development.

## 1. Introduction

In vitro-produced embryos are widely used for assisted reproduction in domestic animals and humans. In the past decades, the quality of in vitro-produced embryos has been improved by modulating the culture conditions for oocyte maturation and embryo development. However, the quality of in vitro- and in vitro-developed embryos remains considerably different, and accounts for a low pregnancy rate, abnormal birth weight, and health risk for the offspring when using in vitro-developed embryos (cows: [1]; humans: [2,3]). The differences are believed to be due to epigenetic alterations caused by improper in vitro culture conditions [4].

After fertilization, the reprograming of the epigenetic landscape is crucial for the establishment of the cellular lineage and proper embryonic development, and is accompanied by global DNA methylation [5]. Global DNA methylation levels are higher for in vitro-produced porcine embryos than for those developed in vivo [6]. Consistently, several studies have reported the differential expression and methylation of genes between in vivo- and in vitro-produced embryos [7,8].

Fertilization and early embryonic development take place in the oviduct, which is filled with oviductal fluid (OF). OF contains a myriad of molecules that modulate the condition of embryo development [9,10]. The supplementation of culture medium with the fluid collected from the oviduct or uterus supports embryonic development [11]. Furthermore, the supplementation of culture medium with OF or extracellular vesicles (EVs) derived from OF alters the methylation levels of several genes [12], reduces global methylation levels, and increases embryonic development [13,14]. EVs contain miRNA, mRNA, and protein in the female reproductive tract, and play a role in cellular communication modulating fertility and embryonic development [15]. miRNA consists of 18–25 nt ribonucleotides, which suppress the stability of mRNAs, presenting a target sequence for miRNAs on 3′-untranslated regions. Aoki et al. [16] reported that miR-17-5p in the EVs of OF with a high frequency supported embryonic development. However, the origin of miRNAs in OF has not been elucidated. These miRNAs could possibly originate from follicular fluid (FF). FF is predicted to flux into the oviduct during ovulation because hormones found at high concentrations in FF were also detected in OF in golden hamsters [17], and hormone and protein concentrations in the ipsilateral oviduct of ovulated ovaries differ from those of their contralateral counterparts [18]. 

Empirical evidence shows that FF contains certain beneficial factors that improve oocyte quality [19,20]. In addition, EVs extracted from FF support cumulus cells expansion and granulosa cell proliferation [21,22]. Embryonic development is supported by the supplementation of the culture medium with EVs isolated from FF [23], and EVs from FF alter the transcriptome profile of bovine epithelial cells [10]. Navakanitworakul et al. [24] showed that lipid bilayers that enclose FF vesicles contain miRNAs. However, which miRNAs play a distinct role in embryonic development and the targets of miRNAs are still unclear.

Here, we demonstrate a novel role of miRNAs in EVs derived from FF in global demethylation and embryonic development.

## 2. Results

### 2.1. EVs in FF Induced the Demethylation of In Vitro-Produced Embryos

**Figure 1:**
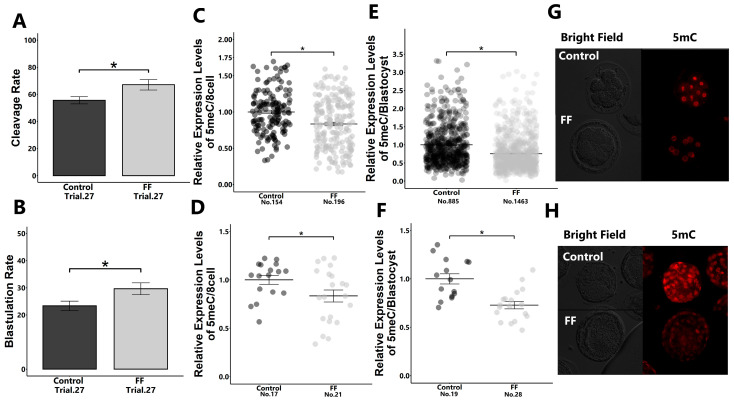
Effect of FF treatment on early embryonic development and 5mC expression levels in embryos. Presumptive zygotes (18 h post-insemination) were cultured with 0% (control) or 1% FF for 30 h, and the cleavage rate of 8-cell-stage embryos (**A**) and blastulation rate (**B**) were determined. Relative expression levels of 5mC in 8-cell-stage embryos ((**C**): blastomere; (**D**): whole embryos) and blastocysts ((**E**): blastomere; (**F**): whole embryos) were determined using immunostaining. (**G**,**H**): Representative images of 5mC in 8-cell-stage embryos (**G**) and blastocysts (**H**). “No.” indicates the number of samples used. Data are presented as mean ± SEM; * *p* < 0.05. Mann–Whitney U test was used for comparison between two groups.

The supplementation of culture medium with 1% FF during the early developmental stage from 18 to 48 h post-insemination significantly increased the developmental rate to the blastocyst stage and reduced global DNA methylation levels (5-methylcytocyne; 5mC) in embryos in both 8-cell- and blastocyst-stage embryos (Figure 1A–H).

On the contrary, 5-hydroxymethylcytosine (5hmC) levels did not differ between the FF-treated and non-treated groups (Figure S1).

**Figure 2:**
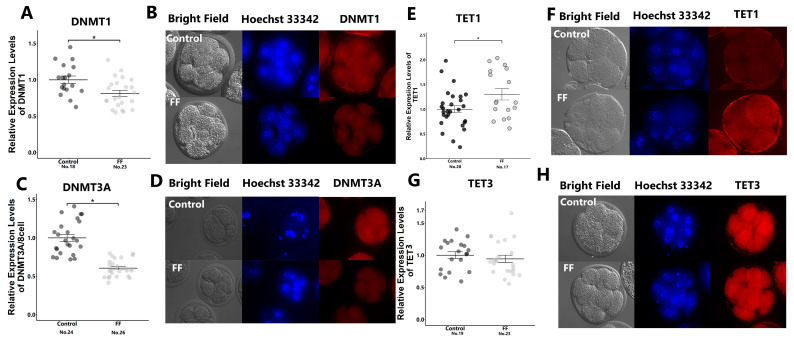
Effect of FF treatment on expression levels of methyltransferases and demethylase in 8-cell-stage embryos. Presumptive zygotes (18 h post-insemination) were cultured with 0% (control) or 1% FF for 30 h, and relative expression levels of DNMT1 (**A**), DNMT3A (**C**), TET1 (**E**), and TET3 (**G**) were determined using immunostaining. (**B**,**D**,**F**,**H**): Representative images of DNMT1 (**B**), DNMT3A (**D**), TET1 (**F**), and TET3 (**H**) in 8-cell-stage embryos. “No.” indicates the number of samples used. Data are presented as mean ± SEM; * *p* < 0.05. Mann–Whitney U test was used for comparison between two groups.

Immunostaining revealed that FF treatment significantly reduced the expression levels of DNMT1, DNMT3A, and TET1, but had no effect on TET3 levels (Figure 2A–H). 

**Figure 3:**
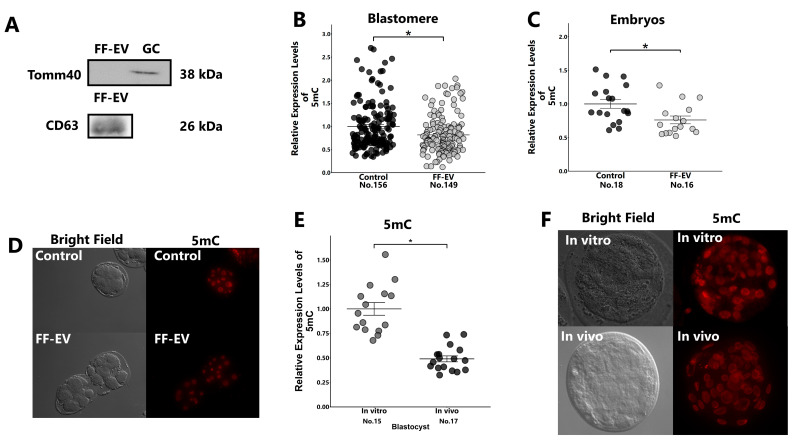
Western blotting of EVs and GCs, and comparison of 5mC expression between EV treated and untreated embryos, or between in vivo- and in vitro-developed blastocysts. (**A**): Protein levels of CD63 and Tomm 40 in EVs and GCs. Presumptive zygotes (18 h post-insemination) were cultured with or without EVs for 30 h, and the expression levels of 5mC in 8-cell-stage embryos ((**B**): blastomere; (**C**): embryos) were examined using immunostaining. In addition, the expression levels of 5mC in blastocysts collected from super-ovulated cows (in vivo) or in vitro-developed blastocysts (in vitro) were examined using immunostaining (**E**). (**D**,**F**): Representative images of 8-cell-stage embryos (**D**) and blastocysts (**F**). “No.” indicates number of samples used. Data are presented as mean ± SEM; * *p* < 0.05. Mann–Whitney U test was used for comparison between two groups.

Using Western blotting, we detected the expression of CD63 (a membrane protein in EVs) but not of Tomm40 (a mitochondrial protein) in the collected EVs (Figure 3A), which indicated that the collected EVs were free of any protein of cellular origin. The 8-cell-stage embryos cultured in the medium containing FF-EVs had low global 5mC levels compared with the non-treated control (Figure 3B–D). In vivo-developed blastocysts had low global DNA methylation levels compared with their in vitro-developed counterparts (Figure 3E,F).

### 2.2. Identification of Molecules Affecting DNA Methylation

**Figure 4:**
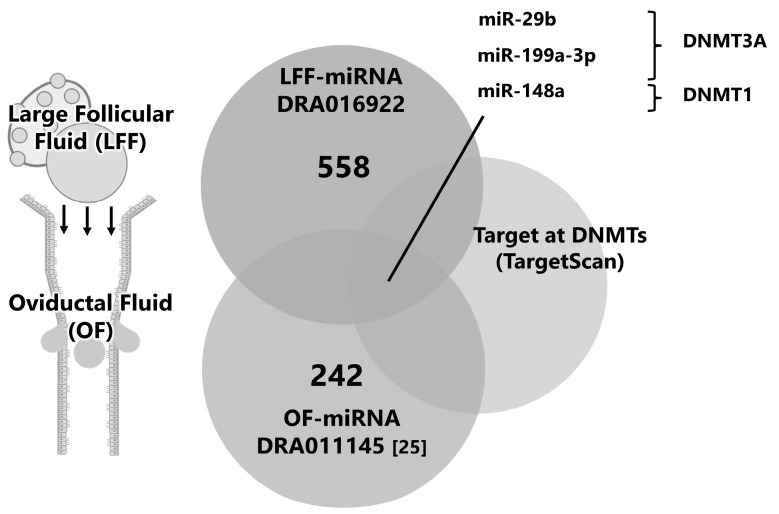
Schematic design for the selection of candidate miRNAs in FF. The miRNAs in EVs derived from large FF (LFF, >10 mm in diameter) were subjected to small RNA-seq and 558 miRNAs were detected (DRA016922). The miRNAs in LFF were compared with the 242 miRNAs in EVs of OF (DRA011145 [25]), and miRNAs targeting DNMTs (in silico prediction using TargetScan, http://www.targetscan.org (accessed on 19 May 2024)).

We explored miRNAs in FF-EVs on the premise that miRNAs in the FF-EVs flux into the oviduct and affect early embryonic development and global demethylation. In a previously reported small RNA-seq analysis, 558 miRNAs were identified in FF from large follicles. By comparing miRNAs with those reported previously from OF (registered in DDBJ as DRA01145 [16]), the common miRNAs between the two groups were selected. After filtering, these miRNAs, based on their targeting of DNMT1 or DNMT3A, were identified. miR-29b, -199a-3p, and -148a are candidate miRNAs for further investigation (Figure 4). 

### 2.3. miRNA Mimic Transfection and Assay of EV Uptake by Embryos

**Figure 5:**
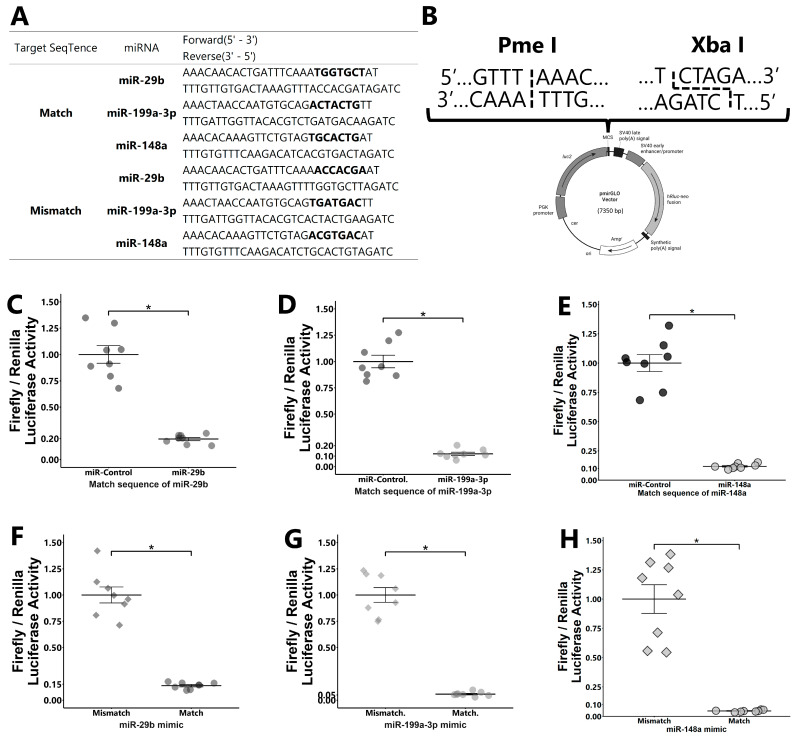
Dual luciferase reporter assay. pmirGLO vectors with oligonucleotides having match or mismatch target sequences of miRNAs (**A**) inserted into PmeI and XbaI sites in the multi-cloning site (**B**) were used. GCs were transfected with the pmirGLO vector with a matched target sequence of miRNAs and then co-transfected with miRNAs ((**C**): miR-29b; (**D**): miR-199a-3p; and (**E**): miR-148a) or control mimic. The expression value for the control was defined as 1.0. GCs were transfected with the pmirGLO vector with matched or mismatched target sequences and then co-transfected with miRNA mimics ((**F**): miR-29b; (**G**): miR-199a-3p; and (**H**): miR-148a). The average value of mismatch was defined as 1.0. Each experiment consisted of 8 replicates. Data are presented as mean ± SEM; * *p* < 0.05. Student’s *t*-test was used for comparison between two groups.

The transfection of miR-29b, -199a-3p, and -148a mimics (Figure 5A) into granulosa cells that were co-transfected with a dual luciferase assay vector having a target sequence (Figure 5B) significantly reduced firefly/Renilla luciferase activity compared with that of their miR-control mimic-transfected counterparts (Figure 5C–E). Furthermore, the transfection of miR-29b, -199a-3p, or -148a into the granulosa cells that were transfected with a Glo-luciferase vector having a respective target sequence reduced the firefly/Renilla luciferase activity compared with that in cells transfected with a Glo-luciferase vector having a mismatch sequence (Figure 5F–H).

**Figure 6:**
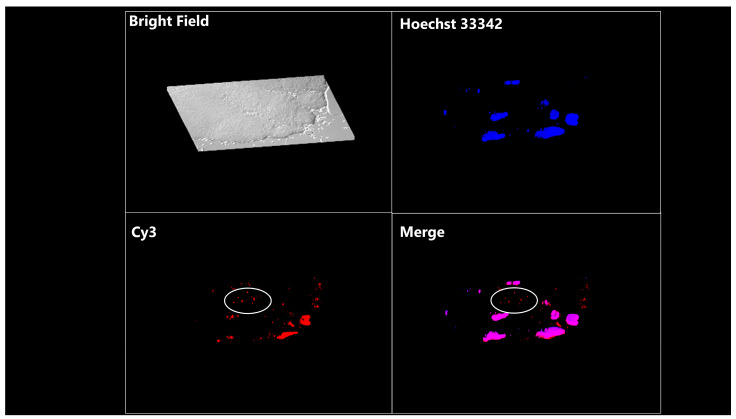
Representative images of blastomeres of 8-cell-stage embryos treated with EV-enclosed Cy3-miRNA. EVs were co-incubated with embryos for 30 h. The image of embryos was reconstructed using the z-stack function (31 stacked images of 0.2 µm thickness captured in the equatorial region) and Leica LAS AF software (version 4.0.0.11706). White circles indicate representative positive signals for red fluorescence.

In addition, after the co-incubation of embryos with EVs containing signal-conjugated miRNAs, intracytoplasmic signals were observed in embryos following a Z-stack observation (×400, Figure 6). 

### 2.4. Transfection of Zygotes with miR-29b, miR-199a-3p, and miR-148a

**Figure 7:**
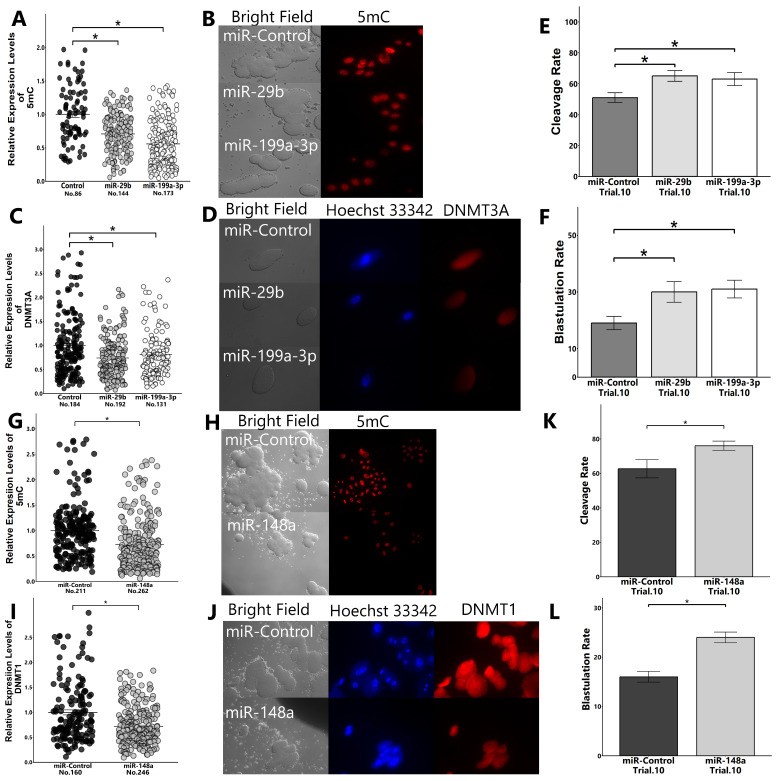
Effect of miRNA transfection on the early embryonic development and expression levels of 5mC and DNMTs. Presumptive zygotes (18 h post-insemination) were cultured with the control mimic or miRNA mimics (miR-29b, -199a-3p, and -148a) for 30 h, and the expression levels of 5mC ((**A**): miR-29b and -199a-3p; (**G**): miR-148a) and DNMTs ((**C**): DNMT3A; (**I**): DNMT1) in 8-cell-stage embryos were examined using immunostaining. The cleave rate to 8-cell-stage embryos ((**E**): miR-29b and -199a-3p; (**K**): miR-148a) and the blastulation rate ((**F**): miR-29b and -199a-3p; (**L**): miR-148a) were determined. (**B**,**H**): Representative images of 5mC in 8-cell-stage embryos ((**B**): miR-29b and -199a-3p; (**H**): miR-148a). (**D**,**J**): Representative images of DNMTs in 8-cell-stage embryos ((**D**): DNMT3A; (**J**): DNMT1). “No.” indicates number of samples used. Data are presented as mean ± SEM, * *p* < 0.05. We used the Mann–Whitney U test between two groups and the Kruskal–Wallis test followed by Steel–Dwass test for comparison among three groups.

The transfection of the embryos with miR-29, -199a-3p, and -148b mimics reduced the expression levels of global 5mC and improved the blastulation rate. Furthermore, these miRNAs reduced the protein levels of targeting DNMTs (Figure 7A–L).

### 2.5. Validation of the Hypothesis That FF Containing High Levels of miR-29b, 199-3p, and 148a Reduce DNA Methylation Levels

**Figure 8:**
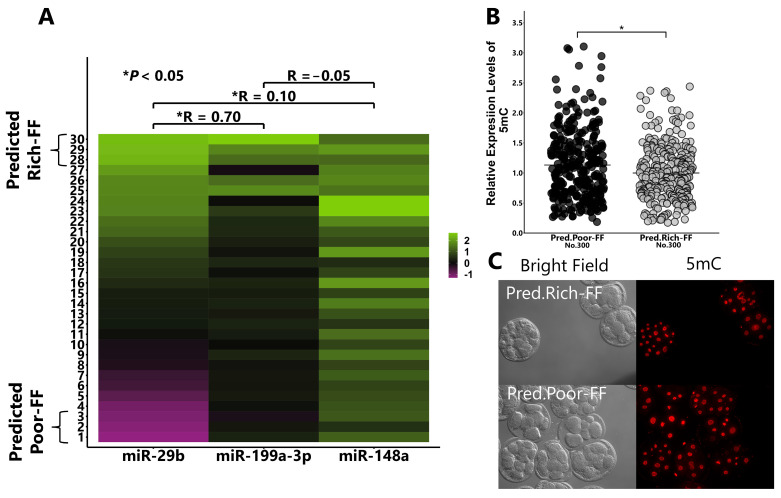
Effect of Predicted Rich-FF or Predicted Poor-FF treatments on the expression levels of 8-cell-stage embryo. Correlation among the contents of miR-29b, -199a-3p, and -148a in the FF of 30 cows (**A**). Based on the contents of miR-29b and -199a-3p in the FF, the top 3 and bottom 3 FFs were defined as Predicted Rich-FF and Predicted Poor-FF, respectively. Presumptive zygotes (18 h post-insemination) were cultured with Predicted Rich-FF or Poor-FF for 30 h, and the expression levels of 5mC in 8-cell-stage embryos were measured using immunostaining (**B**). (**C**): Representative image of 5mC. “No.” indicates number of samples used. Data are presented as mean ± SEM, * *p* < 0.05. the Mann–Whitney U test was used for comparison between two groups.

We collected FF from 30 cows and determined the miRNA concentration using RT-qPCR. The top or bottom three miRNA-rich and -poor FFs were identified and the concentrations of these three miRNAs correlated. Furthermore, the addition of Predicted Rich-FF (top three FF) to the culture medium significantly reduced the 5mC levels compared with the addition of Predicted Poor-FF (bottom three FF) counterparts (Figure 8A–C). 

### 2.6. RNA-seq of FF-Treated 8-Cell-Stage Embryos and Blastocysts

RNA-seq of the 8-cell-stage embryos revealed 1299 DEGs (absolute fold change > 2, *p* < 0.05). Functional annotation analysis revealed that these DEGs were significantly enriched for metabolic pathways, the PI3K-Akt signaling pathway, and carbon metabolism (Table 1). 

Gene ontology (GO) analysis of the DEGs showed a positive regulation of gene expression is the top biological process. Ingenuity pathway analysis predicted the upstream regulators of the DEGs with miR-29b and miR-148. A list of miRNAs is shown in Table S1. On exploring genes associated with methyltransferases and demethylases among the DEGs, DNMT1 (-1.4-fold, *p* < 0.05) and TET1 (4.2-fold, *p* < 0.05) were found to be differentially expressed between the FF-treated and non-treated 8-cell-stage embryos.

RNA-seq of the blastocysts revealed 451 DEGs (absolute fold change > 2.0, *p* < 0.05). DAVID functional annotation showed that significant Kyoto encyclopedia of genes and genomes (KEGG) pathways and GO terms enriched for DEGs included the PI3K-Akt signaling pathway, regulation of transcription, and cellular differentiation. The top pathways and GO terms are listed in Table 2. 

## 3. Discussion

In the present study, we showed that the supplementation of a low concentration of FF in prenuclear 8-cell-stage embryos improves the developmental ability of the zygote, and FF treatment supports the demethylation of embryos through the downregulation of DNMTs. In addition, miR-199a-3p, miR-29b, and miR-148a in the EVs of FF play a role in the beneficial effect of FF. 

The contribution of FF to the embryonic environment has been indicated by the evidence that OF profiles [18] and gene expression in oviductal epithelial cells [10] and hormone concentration in OF [17] differ between ipsilateral and contralateral ovulation sides. Asaadi et al. [26] showed that the size and concentration of EVs are similar in OF and FF in cows; the supplementation of oocyte maturation medium with EVs of FF or OF improved embryonic development. Recently, FF or EVs derived from FF were reported to improve embryonic development [23]. Consistently, in the present study, we observed that FF improves embryonic development, which indicates that components of FF play important roles in developmental events. The demethylation of DNA during early developmental embryos is crucial for proper development [27]. Several studies have shown that 5mC levels differ between in vivo- and in vitro-developed blastocysts [7]. Consistently, we showed low 5mC levels in in vivo-developed blastocysts compared to those in their in vitro-developed counterparts. After fertilization, paternal DNA is demethylated by TET3 and maternal DNA is passively demethylated under a low expression of DNMTs and high TET1 expression [28,29,30,31]. Here, RNA-seq of the 8-cell-stage embryos revealed high TET1mRNA levels and low DNMT1 levels in FF-treated embryos. In addition, protein levels of DNMT1 and DNMT3A were lower and levels of TET1 were higher in the FF-treated embryos. These results suggest that certain molecules inhibit the expression levels of DNMTs and the moderated demethylation conditions. 

IPA of the DEGs in FF-treated 8-cell-stage embryos showed that several miRNAs could have the same directional impact on gene expression with FF treatment. Here, we hypothesized that miRNAs present in FF and OF help demethylation by targeting DNMTs. DEGs are known to be associated with the metabolic pathway and carbon metabolism. Carbon metabolism is important for DNA methylation because it provides a methyl donor and substrate for TET3 activity [32,33]. Consistently, the pyruvate metabolic process is the top GO term and its metabolites, such as acetyl CoA and α-ketoglutarate, are crucial for zygotic genome activation [34]. However, in the present study, we did not examine the reason for high TET1 expression and the significance of altered embryonic metabolism. The present study showed that FF treatment increased TET1 expression in 8-cell-stage embryos; TET1 expression was shown to increase in cows [35] and pigs [36]. The 5hmC levels did not differ between FF-treated and non-treated embryos; however, TET1 hydroxylated 5mC to 5hmC, 5-formylcytosine (5fC), and 5-carboxylcytosine (5caC), subsequently. Further experiments are required to determine whether TET1 plays an important role in the demethylation of early developmental-stage embryos.

FF contains a myriad of molecules, which potentially affect embryonic development. Among these, EVs affect not only oocyte maturation, but also embryonic development [19]. The supplementation of culture medium with EVs extracted from FF also reduced the levels of 5mC in embryos, indicating that EVs play a role in FF-induced demethylation. In addition, the fact that embryos could uptake miRNAs contained in EVs derived from GCs indicates that miRNAs in FF could affect early embryonic development. Matching small RNA-seq data (miRNAs in FF and OF) with their targets, including DNMTs, led to the identification of miR-29b, -199a-3p, and -148a as candidate players in the demethylation of early developmental-stage embryos. These miRNAs were also found in bovine GCs (DRA013126) and in the FF of middle-sized follicle-derived FF (DRA011147). These miRNAs are present in FF throughout oocyte maturation and can contribute to the miRNA profiles in OFs. Interestingly, RT-qPCR of miRNAs in EVs of FF showed a high correlation between miR-29b and -199a-3p. Consistently, in our recent study, good-quality follicle-associated miR-151-3p and -425-5p were correlated; however, miR-151-3p and -148a were not significantly correlated [25]. Based on these findings, we suggest that certain groups of miRNAs are actively secreted from GCs through a selective packaging system. 

miR-29b is important for embryonic development in pig parthenogenetically activated embryos [37]. Moreover, miR-29 suppresses DNMTs to improve embryonic quality [37]. In addition, a high amount of miR-199a in spent culture medium and circulation were reported to be associated with pregnancy outcomes [38,39], and the suppression of miR-199a reduced the developmental ability of mouse embryos [40]. On the contrary, miR-148 is the most abundant miRNA in FF, but its effect on embryonic development has not been reported. In the present study, these miRNAs reduced the levels of target proteins in the embryos and increased the developmental rate to the blastocyst stages. These results suggest that miR-29b, -199a-3p, and-148a induce demethylation and embryonic development, and miRNAs in FF are a possible resource. In line with this notion, we show that FF, containing high concentrations of miR-29b and -199a-3p, has a high demethylation ability. However, the optimal concentration of miRNAs remains unclear, warranting further research.

Considering that in vitro-developed blastocysts differ from those developed in vivo, it is pertinent to investigate whether FF treatment affects gene expression in embryos. The top GO terms included the regulation of transcription from RNA polymerase II, trophoblast differentiation, and zygotic genome activation closely linked to polymerase II binding [41,42]. We performed RNA-seq using in vivo- and in vitro-developed embryos, which used the same cryopreserved semen lot and the embryo culture protocol for blastocyst production (DRA006210 [8]). Using the differentially expressed genes, the top GO terms included the regulation of transcription from RNA polymerase II. High activity of PI3K-Akt signaling is associated with the quality of the bovine embryo [43]. However, further experiments are needed to evaluate the long-term consequence of the demethylation state of blastocysts.

In conclusion, the presence of the components of FF, such as miR-29b and miR-199a-3p, during early embryonic development plays a role in supporting development and DNA demethylation.

## 4. Materials and Methods

### 4.1. Chemicals and Medium

All chemicals were purchased from Nacalai Tesque (Kyoto, Japan), unless otherwise described. The medium used for bovine granulosa cell (GC) incubation (GC culture medium) was medium 199 (Gibco, Grand Island, NY, USA) supplemented with 5 mM taurine, antibiotics, and 5% fetal calf serum (FCS; 21B00A; Nichirei Biosciences Inc., Tokyo, Japan). For the miRNA transfection of GCs, FCS was replaced with exosome-free fetal bovine serum (Exo-FBS; System Biosciences, Palo Alto, CA, USA). The medium used for the in vitro maturation (IVM) of oocytes was medium 199 supplemented with 10% FCS, 5 mM taurine, and antibiotics. The media used for in vitro fertilization (IVF) and in vitro culture (IVC) were prepared based on synthetic oviductal fluid (SOF), as described previously [16]. The IVC medium used from the presumptive zygote to 8-cell-stage embryos contained 1% FCS; in the case of the co-incubation of EVs and/or transfection of embryos with miRNA mimics, the FCS was exchanged with Exo-FBS. The IVC medium used for embryo culture from the 8-cell stage to the blastocyst stage contained 5% FCS. The incubation of bovine GCs and oocytes (IVM and IVF) was conducted under atmospheric conditions of 5% CO_2_ and 95% air at 38.5 °C. The IVC of embryos was conducted under atmospheric conditions of 5% CO_2_, 5% O_2_, and 90% air at 38.5 °C. 

### 4.2. Oocyte Collection, IVM, IVF, and IVC

Ovaries were collected from Japanese Black cows at a local slaughterhouse, stored at approximately 25 °C in phosphate buffered saline (PBS) containing antibiotics, and transferred to the laboratory within 4 h. Cumulus cell–oocyte complexes (COCs) and GCs were aspirated from antral follicles (3–6 mm in diameter) using an 18 G needle (Terumo, Tokyo, Japan) connected to a 10 mL syringe (Terumo). COCs were collected from follicular contents under a stereomicroscope (Olympus, Tokyo, Japan) and were subjected to IVM, IVF, and IVC. 

### 4.3. Preparation of FF

Ovaries from individual cows from an identical herd were collected, and the corpus luteum was classified based on a previous report [44]. Twenty cows with a mature corpus luteum in the ovary were selected (stage III, predicted to be 11–17 days post-ovulation). FF was aspirated from antral follicles (3–6 mm in diameter) from at least 20 cows and equally mixed to create FF batches to reduce individual differences. A total of three batches of FF (total of 60 cows) were prepared. We identified preovulatory cows based on the presence of mucus around the cervix, normal large follicles (>10 mm), and a regressed corpus luteum. The cows were rare, but we collected large FF (LFF) from three cows. The FF was centrifuged at 4500× *g* for 10 min at 25 °C to remove cellular debris; the supernatants were filtered through a 0.20 μm pore filter (Minisart, Sartorius Stedim Biotech GmbH, Goettingen, Germany) to remove vesicles larger than 200 nm. The FF was preserved at −80 °C until it was used for experiments.

### 4.4. Effect of Supplementation of IVC Medium with FF on Embryonic Development

Eighteen hours after insemination, presumptive zygotes were denuded from the surrounding cells and allocated to IVC medium containing 0 or 1% FF and incubated for 30 h. FF concentration was determined in a pre-experiment wherein the effects of 0.1% and 1% FF on the demethylation levels of embryos were examined. FF was randomly selected from the three batches of FF. The cleavage rate to the 8-cell-stage embryos was examined, and they were subsequently incubated in the IVC medium for 5 days when the blastulation rate was determined. The incubation experiment was repeated 27 times.

### 4.5. Immunostaining

Embryos were fixed in 4% paraformaldehyde, followed by permeabilization in Triton-X 100 (0.25%) for 30 min. The embryos were blocked in PBS containing 5% BSA for 1 h. For 5mC staining, the embryos were treated with 1N HCl for 1 h before blocking. Subsequently, these samples were incubated with primary antibodies (×200 dilutions) (rabbit anti-DNMT1 (60B1220.1, NOVUS, Chesterfield, MO, USA), DNMT3A (AI12955, ABCEPTA, San Diego, CA, USA), TET3 (GTX121453, GeneTex, Irvine, CA, USA), 5mC (D3S2Z, Cell Signaling, Danvers, MA, USA), or mouse anti-5hmC (GTX629701, GeneTex) overnight), followed by treatment with a secondary antibody (×500 dilutions) (anti-rabbit IgG (H+L), 1:500, F(ab’)2 fragment (Alexa Fluor^®^ 488 conjugate), Cat# 4412). The embryos were mounted on a glass slide using antifade (SCR-038447, Dianova, Hamburg, Germany) and observed, and images were captured under a fluorescence microscope (DMI 6000 B; Leica, Wetzlar, Germany). To determine the fluorescence intensity of DNMT1/3A and TET1/3, an image of the equatorial region of embryos was evaluated using ImageJ (ver. 1.54d, NIH, Bethesda, MD, USA). To determine the fluorescence intensity of 5mC and 5hmC, each nucleus (without an overlapped blastomere) was selected and the fluorescence intensity was measured using ImageJ (NIH).

### 4.6. Isolation of EVs from FF and the Extraction of miRNAs from EVs

EVs were isolated from FF and LFF using ExoQuick (EXOQ20A-1, System Biosciences), according to the manufacturer’s instructions. Each 500 µL FF and LFF was mixed with ExoQuick and incubated overnight at 4 °C followed by centrifugation (15,000× *g* for 2 min) to obtain a pellet of EVs. EVs collected from FF and LFF were used for RNA extraction. miRNAs in the EVs were extracted with a SeraMir Exosome RNA Amplification Kit (System Biosciences, RA806A-1) and used for qRT-PCR or small RNA-seq. EVs isolated from FF were used for Western blotting analysis or culture experiments. EVs diluted in PBS (500 µL) were added to the culture medium at a concentration of 1%. The kit used for EV preparation confirmed CD63 expression and the absence of mitochondrial proteins [44]. We also validated the presence of CD63 in and absence of Tomm 40 from EVs.

### 4.7. Collection and Preparation of Live Granulosa Cells

Follicular contents were aspirated from antral follicles (3–6 mm in diameter), as described above. After the collection of COCs, the remaining follicular contents were filtered through a 75 μm cell strainer to remove cellular debris, followed by centrifugation at 200× *g* for 1 min to obtain a cellular pellet. The GCs were incubated overnight in medium 199 containing 5% FCS in a culture plate (90 mm, Sarstedt Inc., Sarsted, Germany). Live GCs attached to the culture plate were collected using Accumax (Innovative Cell Technologies, San Diego, CA, USA). The live GCs were used for Western blotting analysis, transfection of the GLO vector, and miRNA transfection.

### 4.8. Western Blotting

Proteins extracted from EVs and GCs were used for Western blotting analysis. The details of the protocol are presented in the Supplementary Materials and Methods.

### 4.9. Superovulation and Flushing of Embryos 

Three Japanese Black cows were used for in vivo embryo production. The same cryopreserved semen lot was used for in vivo embryo production. The protocol for super ovulation was described by Noguchi et al. [8], and detailed information is presented in the Supplementary Materials and Methods. 

### 4.10. Small RNA Sequences of miRNAs

RNA extraction was performed as described above. After sequencing, data processing and transcriptome analysis were performed using the CLC Genomics Workbench 21.0.3 (Qiagen, Hilden, Germany). The raw data were confirmed to be of a high quality after discarding adapter sequences, ambiguous nucleotides, and low-quality sequences. The data were filtered to identify sequences in the range of 18–25 nt (the length of mature miRNAs) and mapped against miRbase v22 (https://www.mirbase.org/ (accessed on 19 May 2024)) using CLC quantifying miRNA tools to obtain total count sequence reads. Sequence data of miRNAs in LFF were registered in the DDBJ Read Archive under accession number DRA016922. The detailed settings for RNA-seq and small RNA procedures are presented in the Supplementary Materials and Methods.

### 4.11. Preparation of the pmirGLO Vector and miRNA Mimics

Oligonucleotides, including matched and mismatched target sequences of each miRNA (miR-29b, -199a-3p, and -148a), are shown in Figure 5A. These sequences were inserted downstream of the firefly luciferase gene in the pmirGLO Dual luciferase miRNA target expression vector (Promega, Madison, WI, USA) between PmeI/XbaI sites (Figure 5B). The miRNA control mimic and miR-29b, -199a-3p, and -148a mimics were purchased from miRVana (Ambion, Grand Island, NY, USA; details of the sequence are described in Table S2).

### 4.12. Dual Luciferase Reporter Assay

For the dual luciferase assay, live GCs (see Section 4.7) were incubated overnight in a 96-well plate (BD Biosciences, Franklin Lakes, NJ, USA) at a concentration of 2.0 × 10^4^ cells/well. Thereafter, the cell viability and growth (70% confluent) were checked under a microscope, and the cells were used for transfection. Transfection was conducted using Lipofectamine^®^ 3000 (Invitrogen, Carlsbad, CA, USA) and a selective combination of miRNA mimics (final concentration, 60 nM) and pmirGLO vector in the transfection medium of GCs. Two days after the transfection, the luciferase activity of the cells was analyzed using the Dual-Glo^®^Luciferase Assay System (Promega) and a luminometer (Spark 10M; Tecan Japan Co., Ltd., Kanagawa, Japan), according to the manufacturer’s instructions. Firefly luciferase activity was normalized by Renilla luciferase activity.

### 4.13. Preparation of Cy3-Labeled miRNA Mimic 

To check if EVs containing miRNAs derived from GCs can enter embryos, miRNA control mimics were labeled with Cyanine dye 3 (Cy3) using a Label IT siRNA Tracker Cy3 Kit (Mirus, Madison, WI, USA), according to the manufacturer’s instructions. Live GCs (see Section 4.7) were cultured in a 4-well plate at a concentration of 2.0 × 10^5^ cells/well and transfected with the Cy3-labeled miRNA control mimic (Cy3-miRNA, 60 nM) using Lipofectamine^®^ 2000 (Invitrogen) and a transfection medium. A day after lipofection, the GCs were washed, and the medium was exchanged with fresh transfection medium. After 24 h of incubation, the medium was collected and 5 mL of medium was centrifuged (700× *g* for 5 min), followed by filtration through a 0.20 μm pore filter (Minisart). EVs in the medium (5 mL) were isolated using ExoQuick-TC (EXOTC10A-1, System Biosciences) and diluted in PBS (500 μL). The EVs (1% in PBS) were used for the EV-uptake assay.

### 4.14. EV-Uptake Assay

Zona pellucida (ZP) intact presumptive zygotes (18 h post-insemination) were co incubated with EVs containing Cy3-miRNA for 30 h. After incubation, 8-cell-stage embryos were collected and fixed overnight in 4% paraformaldehyde. Before fixation, the ZP of the embryos was removed using 0.5% proteinase (Sigma Aldrich, Saint Louis, MO, USA) to obtain a clear image of Cy3 signals. The fixed embryos were stained with Hoechst 33342 (1 μg/mL) in PBS and then mounted on glass slides with Immunoselect Antifading Mounting Medium (SCR-038447, dianova, Hamburg, Germany). The equatorial regions of the embryos were observed under a fluorescence microscope (DMI 6000 B; Leica, Wetzlar, Germany) to examine inner cellular Cy3 signaling.

### 4.15. Effect of Supplementation of IVC Medium with miRNA Mimics on Embryonic Development

ZP is reported to block the lipofection of siRNAs [45]. Therefore, presumptive zygotes (18 h post-insemination) were treated with 0.5% proteinase (Sigma Aldrich) to remove ZP, and the embryos were then subjected to lipofection, as described previously [16]. The concentration of miRNA mimics was set at 60 nM following the pre-experiment wherein the transfection efficiency was examined using the BLOCK-iT™ Alexa Fluor™ Red Fluorescent Control (14750-100, Invitrogen). The transfection of embryos was conducted by incubation with Lipofectamine^®^ 2000 (Invitrogen) and miRNA mimics (miR-29b, -199a-3p, or -148a) in transfection medium for 30 h. Culture experiments were repeated 20 times. 

### 4.16. Selection of FF Based on the miRNA Content Using RT-qPCR

FF was individually collected from 30 cows and rated based on the miRNA content. The miRNA contents in EVs of each FF was measured using RT-PCR. cDNA was prepared using the Mir-X miRNA RT-qPCR TB Green kit (Takara Bio, Siga, Japan) and PCR was conducted according to the manufacturer’s instructions, including primer sets targeting each miRNA (sequences of miR-29b, -148a, and -199a-3p were obtained from miRbase) and adaptor sequence. The top 3 and bottom 3 FF samples were defined as Predicted Rich-FF and Predicted Poor-FF, respectively, using concentrations of miR-29b and-199a-3p, because these two miRNAs were closely correlated. Predicted Rich-FF and Predicted Poor-FF were added to the culture medium (at a concentration of 1%) to test the hypothesis that miR-29b and -199a-3p contents in FF determine the demethylation ability.

### 4.17. RNA-seq and Analysis of the Data

In vitro-developed 8-cell-stage embryos and blastocyst-stage embryos were subjected to RNA-seq analysis. Embryos were cultured in 0 or 1% FF containing the IVC medium from zygote- (18 h post-insemination, pronuclear stage) to 8-cell-stage embryos (48 h post-insemination) for 30 h. Approximately 50 FF-treated or non-treated 8-cell-stage embryos were used for RNA extraction. In addition, 30 blastocysts developed from the FF-treated or non-treated 8-cell-stage embryos were used for RNA extraction. The details of RNA-seq are described in the Supplementary Materials and Methods. Three samples were prepared for each group using different batches of ovaries. Pathways and GO analysis of the DEGs was conducted using a functional annotation tool (DAVID, https://david.ncifcrf.gov/ (accessed on 19 May 2024))), where Bos taurus was used as the background species. Raw RNA-seq data of 8-cell-stage embryos and blastocysts were registered in DDBJ under the accession numbers DRA017489 and DRA017490.

### 4.18. Publicly Available Database of miRNAs in Bovine FF and OF 

miRNAs in the EVs of FF derived from middle-sized FF and Evs of OF were downloaded from publicly available DDBJ (DRA011147 and DRA011145, respectively). With regard to the miRNA data for the EVs of FF, FF was collected from middle-sized (3–6 mm in diameter) the ovary derived from of 21–40-month-old young Japanese Black cows (almost the same as the present study). miRNAs in the EVs of OFs: OFs were collected from whole oviducts of four Japanese Black cows whose ovaries were predicted as being 2–3 days post-ovulation based on the morphology of the corpus luteum. These data were processed and mapped as described above.

### 4.19. Experimental Design

**Figure 9:**
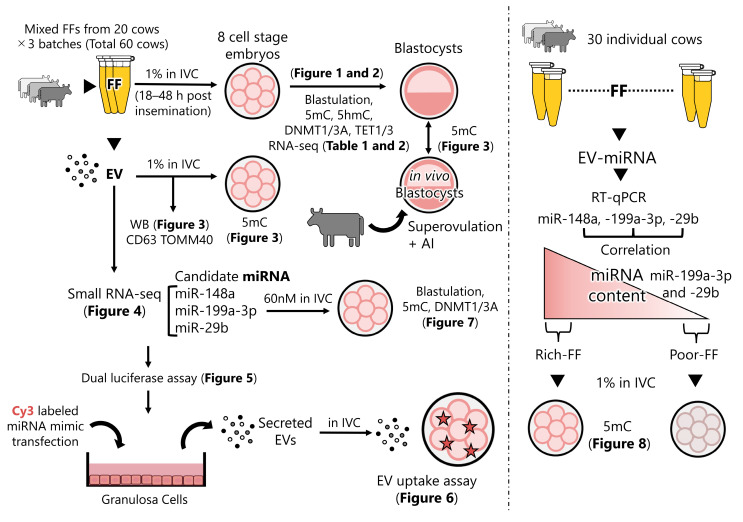
Schematic design of experiments and the respective results. FF of 20 cows was mixed and added to IVC medium (1%) 18–48 h post-insemination, and then the blastulation rate, levels of 5mC, 5hmC, DNMTs, and TETs were examined. The 8-cell-stage embryos were subjected to RNA-seq. Data are presented in Figure 1 and Figure 2 and Table 1 and Table 2. Subsequently, EVs were extracted from the FF; contents of CD63 and TOMM40 in the EVs were examined (Figure 3). The effect of the addition of EVs to the culture medium on the expression levels of 5mC was examined. The levels of 5mC in blastocysts developed in vitro (control) was compared with those of blastocysts developed in vivo (Figure 3). Using small RNA-seq and previous small RNA-seq data, candidate miRNAs were predicted (Figure 4). The dual luciferase assay was used to determine if the miRNA mimic actually targeted the predicted sequence in bovine GCs (Figure 5). The GCs were transfected with miRNA mimics labeled with Cy3. EVs were collected from the culture medium and were then added to the culture medium of embryos to see if the EVs containing miRNAs were taken up by the embryos (Figure 6). The effect of the supplementation of the embryo culture medium with the candidate miRNAs (miR-29b, -199a-3p, and -148a) on the expression of targeted DNMTs was examined. The hypothesis that FF contains miR-29b, -199a-3p, and -148a, which have a strong ability to demethylate DNA in embryos, was tested using FF collected from 30 individual cows (Figure 8).

Figure 9 elucidates the design of the experiment, and the respective figures and tables present the corresponding results.

### 4.20. Statistical Analysis

Data are presented as the mean ± standard error of the mean (SEM). Data were analyzed using the Shapiro–Wilk test for determining normality. For a comparison between the two groups, we used the Student’s *t*-test for data with a normal distribution and the Mann–Whitney U test for data with a non-normal distribution. For a comparison among the three groups, we used the Kruskal–Wallis test followed by the Steel–Dwass test. Statistical significance was set at *p* < 0.05.

## Figures and Tables

**Table 1:** Top KEGG pathways and GO terms for DEGs in FF-treated 8-cell stage embryos.

KEGG Pathway	Count	*p*-Value
Metabolic pathways	76	1.0 × 10^4^
PI3K-Akt signaling pathway	20	3.0 × 10^2^
Carbon metabolism	10	8.0 × 10^3^
Lysosome	10	3.0 × 10^2^
Biosynthesis of amino acids	9	2.0 × 10^3^
Glucagon signaling pathway	9	1.0 × 10^2^
Serotonergic synapse	9	3.0 × 10^2^
Arachidonic acid metabolism	8	2.0 × 10^2^
Starch and sucrose metabolism	5	2.0 × 10^2^
African trypanosomiasis	5	5.0 × 10^2^
GO: Biological process	Count	*p*-value
positive regulation of gene expression	31	1.0 × 10^3^
Cell adhesion	26	1.0 × 10^3^
Lipid metabolic process	21	8.0 × 10^5^
Negative regulation of gene expression	21	4.0 × 10^3^
Defense response to virus	19	2.0 × 10^2^
Positive regulation of cell migration	18	5.0 × 10^3^
Proteolysis	18	1.0 × 10^2^
Homophilic cell adhesion via plasma membrane adhesion molecules	15	3.0 × 10^3^
Axon guidance	14	3.0 × 10^2^
Phosphorylation	13	2.0 × 10^2^

Pathways and GO terms (biological process) were enriched for differential expressed genes (DEGs) in FF-treated 8-cell stage embryos using the DAVID functional annotation tool.

**Table 2:** Top KEGG pathways and GO terms for DEGs in FF-treated blastocysts.

KEGG Pathway	Count	*p*-Value
Pathways in cancer	19	2.0 × 10^2^
PI3K-Akt signaling pathway	14	4.0 × 10^2^
Oxytocin signaling pathway	10	3.0 × 10^3^
Cholinergic synapse	9	2.0 × 10^3^
Transcriptional misregulation in cancer	9	4.0 × 10^2^
Phospholipase D signaling pathway	8	3.0 × 10^2^
Efferocytosis	8	4.0 × 10^2^
GO: Biological process	Count	*p*-value
Regulation of transcription from RNA polymerase II promoter	42	8.0 × 10^3^
Negative regulation of transcription from RNA polymerase II promoter	25	2.0 × 10^3^
Positive regulation of transcription from RNA polymerase II promoter	25	1.0 × 10^2^
Cell differentiation	19	5.0 × 10^3^
Activation of GTPase activity	7	1.0 × 10^2^
SMAD protein signal transduction	6	9.0 × 10^3^
Lipid transport	6	1.0 × 10^2^
Positive regulation of endothelial cell migration	5	1.0 × 10^2^
MAPK cascade	5	3.0 × 10^2^
Cellular response to cAMP	4	3.0 × 10^2^

Pathways and GO term (biological process) were enriched for differential expressed genes (DEGs) in FF-treated blastocysts using the DAVID functional annotation tool.

## Data Availability

All datasets generated from RNA-seq and small RNA-seq have been deposited in DDBJ.

## Supplementary Materials

The following supporting information can be downloaded at: https://www.mdpi.com/article/10.3390/ijms25115872/s1.
