# Supplementary material for: miRNAs in Follicular and Oviductal Fluids Support Global DNA Demethylation in Early-Stage Embryos
Source: Int J Mol Sci. 2024 May 28;25(11):5872. doi: 10.3390/ijms25115872 (PMC11172648; doi:10.3390/ijms25115872)
Supplement: Supplementary file 1 [file ijms-25-05872-s001.zip › Supplementary Figure S1.pdf]

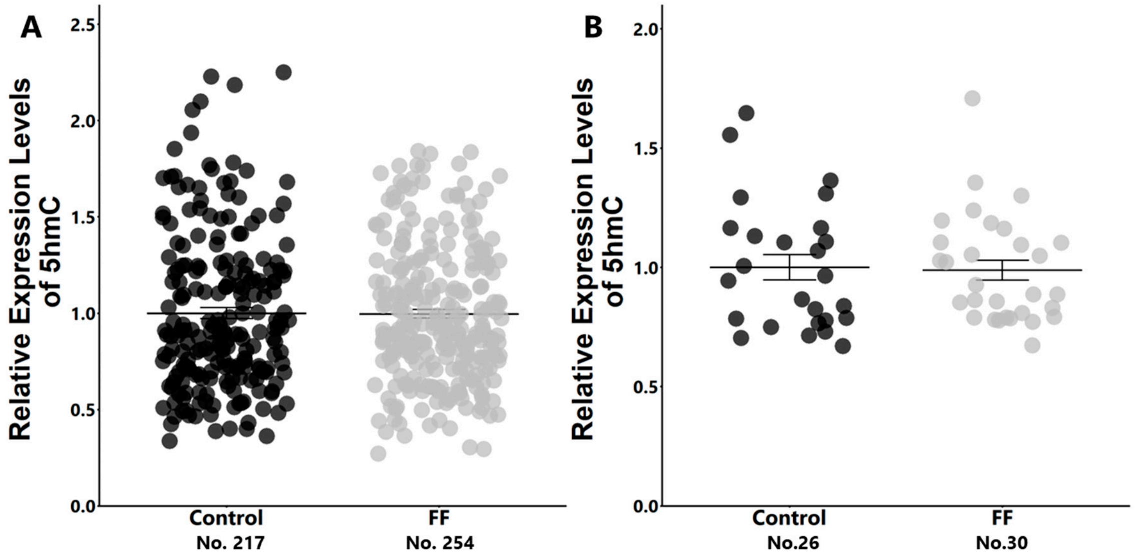


Supplementary Figure S1

Expression levels of 5-hydroxymethylcytosine (5hmC) in FF-treated or non-treated 8-cell stage embryos. Presumptive zygotes (18 h post-insemination) were cultured with 0% (control) or 1% FF for 30 h, and expression levels of 5hmC in the 8-cell stage embryos (A: blastomere, B: whole embryos) were examined using immunostaining. “No.” indicates Data are presented as mean ± SEM.
