# Supplementary material for: miRNAs in Follicular and Oviductal Fluids Support Global DNA Demethylation in Early-Stage Embryos
Source: Int J Mol Sci. 2024 May 28;25(11):5872. doi: 10.3390/ijms25115872 (PMC11172648; doi:10.3390/ijms25115872)
Supplement: Supplementary file 1 [file ijms-25-05872-s001.zip › Supplementary Materials and Methods.pdf]

Supplementary materials and methods

Western blotting

The extracellular vesicles (EVs) and granulosa cells (GCs) were treated with cell lysis buffer (Complete Lysis-M; Roche, Basel, Switzerland) containing protease (Complete protease inhibitor cocktail; Roche) and phosphatase (PhosSTOP; Roche) inhibitors, and were frozen at –80°C until further use. Protein concentration was determined using a Pierce^{TM} BCA Protein Assay Kit (Thermo Fisher Rockford, IL, USA), and 50 μg of protein was co-incubated with Laemmli sample buffer containing 2-mercaptoethanol at 95°C for 5 min. The proteins were separated on 12% sodium dodecyl surface-polyacrylamide gels and transferred onto polyvinylidene fluoride membranes (Trans-Blot Turbo Mini Transfer Packs; Bio-Rad, CA, USA) using the Trans-Blot Turbo Transfer System (Bio-Rad). The primary antibodies used were goat anti-CD63 (orb11597; Biorbyt, Cambridge, UK) or rabbit anti-Tom40 (sc-11414; Santa Cruz Biotechnology, CA, USA). Donkey anti-goat IgG(H+L), horseradish peroxidase (HRP) conjugate (A15999; Life technologies, MD, USA) or HRP donkey anti-rabbit antibodies (ab6802; Abcam, Cambridge, UK) were used as secondary antibodies.

Superovulation and flushing of embryos

Cows with corpus luteum were inserted with a progesterone-releasing intravaginal device (controlled internal drug release (CIDR), Livestock Improvement Corporation, Tokyo, Japan). The day of CIDR insertion was defined as day 0. On day 1, 2mg estradiol benzoate (Asuka, Tokyo, Japan) was administered to cows, and on day 6. 20 AU FSH (Antrin, Kyoritsu, Tokyo, Japan) was administrated for three days. On the morning of day 8, the cows were treated with PGF2α (d-cloprostenol, Dalmazin, Kyoritsu, Tokyo, Japan), and the CIDR was removed. Two days after the PGF2α treatment, artificial insemination was performed. Frozen-thawed semen used for artificial insemination is the same lot of the semen bull, which was used for other experiments. The resulting embryos were non-surgically

flushed from the uterus at 6.5 days after insemination. The embryos were classified using the IETS manual and only embryos categorized as grade 1 were used for experiments.

Small RNA-seq analysis

RNA quality and concentration were examined using a Bioanalyzer (Agilent Technologies, Palo Alto, CA, USA). A cDNA library of RNAs from follicular fluid (FF) was constructed using an Illumina SMARTer® smRNA-Seq Kit for Illumina® (Illumina, San Diego, CA, USA). The average length of all derived libraries was confirmed using an Agilent Bioanalyzer with a High Sensitivity DNA Kit (Agilent Technologies, Palo Alto, CA, USA), and the concentration of each library was adjusted to 10 nM based on qPCR results (KAPA Biosystems, Boston, MA, USA). The multiplexed sample was sequenced as 75 single-read cycles on a NextSeq 500 system (Illumina). Image analysis, base calling, and quality filtering were performed using bcl2fastq2 v2.18.0.12 (Illumina), following the manufacturer's instructions. Sequence preparation and mapping to the reference genome were performed on the CLC Genomics Workbench ver. 22.0.2 (Qiagen, Hilden, Germany).

RNA-seq analysis

RNA quality and concentration were examined using the Agilent 2100 Bioanalyzer (Agilent Technologies, Palo Alto, CA, USA). cDNA was produced using the NEBNext Single Cell/Low Input RNA Library Prep Kit (New England Biolabs, MA, USA). Quality and quantity of cDNA were determined using the Agilent 2100 Bioanalyzer, followed by re-measurement using the Kapa Library Quantification Kit (Kapa Biosystems, MA, USA). Sequencing was performed using NextSeq1000 (Illumina, CA, USA) single read × 100 bp. Image analysis, base calling, and quality filtering were performed using the RTA version 2.4.11 (Illumina), following the manufacturer's protocol, and the sequence data were converted to Fastq using bcl2fastq2 v2.20.0.422. Sequence data were filtered to

discard adapter sequences, ambiguous nucleotides, and low-quality sequences. The average total number of sequence reads for each sample was 25,566,579.0 ± 378,582.9 for 8-cell stage embryos and 25,566,579.3 ± 378,582.9. The remaining sequence data were aligned to the *Bos taurus* genome sequence (ARS-UCD1.2/bosTau9) to count the sequence reads. Gene expression values were evaluated using transcript per kilobase million (TPM). Sequence preparation, mapping to the reference genome, and differential gene expression analysis (absolute difference > 2, and $P < 0.05$) were performed on the CLC Genomics Workbench ver. 22.0.2 (Qiagen, Hilden, Germany).
