# Supplementary material for: miRNAs in Follicular and Oviductal Fluids Support Global DNA Demethylation in Early-Stage Embryos
Source: Int J Mol Sci. 2024 May 28;25(11):5872. doi: 10.3390/ijms25115872 (PMC11172648; doi:10.3390/ijms25115872)
Supplement: Supplementary file 1 [file ijms-25-05872-s001.zip › Supplementary Table S1.pdf]

Table S1. Upstream regulator predicted using DEGs of FF-treated 8 cell stage embryos		
Upstream Regulator	Molecule Type	p-value
miR-181a-5p	mature microRNA	4.E-02
miR-200b-3p	mature microRNA	1.E-02
miR-27a-3p	mature microRNA	4.E-02
miR-186-5p	mature microRNA	1.E-02
miR-204-5p	mature microRNA	7.E-05
miR-141-3p	mature microRNA	4.E-02
let-7a-5p	mature microRNA	8.E-03
miR-375-3p	mature microRNA	7.E-03
miR-143-3p	mature microRNA	3.E-02
miR-338-3p	mature microRNA	4.E-02
miR-1-3p	mature microRNA	2.E-02
miR-29b-3p	mature microRNA	2.E-02
miR-9-5p	mature microRNA	3.E-03
miR-16-5p	mature microRNA	1.E-04
miR-182-5p	mature microRNA	1.E-02
miR-124-3p	mature microRNA	1.E-02
miR-122-5p	mature microRNA	1.E-02
miR-199a-5p	mature microRNA	3.E-02
miR-140-5p	mature microRNA	1.E-02
miR-508-3p	mature microRNA	4.E-02
mir-802	microRNA	9.E-04
mir-183	microRNA	1.E-02
mir-148	microRNA	6.E-03
mir-27	microRNA	4.E-03
mir-15	microRNA	1.E-02
mir-29	microRNA	8.E-03
mir-103	microRNA	2.E-02
mir-182	microRNA	9.E-03
mir-34	microRNA	2.E-02
mir-137	microRNA	3.E-02
mir-122	microRNA	1.E-02
mir-140	microRNA	7.E-03
mir-340	microRNA	2.E-02

mir-204	microRNA	5.E-02

Significant upstream regulator predicted using Ingenuity Pathway Analysis (IPA) software.
